# Dyadic Loneliness and HbA1c Levels Among Older Married Couples: Evidence From The Irish Longitudinal Study on Ageing

**DOI:** 10.1093/geroni/igab046.2783

**Published:** 2021-12-17

**Authors:** Jeffrey Stokes, Adrita Barooah

**Affiliations:** 1 University of Massachusetts Boston, Boston, Massachusetts, United States; 2 UMass Boston, Boston, Massachusetts, United States

## Abstract

Loneliness is an important determinant of health and mortality among the aging population, including for cardiometabolic health. Yet research has largely focused on individual experiences of loneliness, rather than taking intimate relationships into account. However, recent studies have highlighted that the psychosocial well-being of one’s partner may impact one’s own health as well. Indeed, the stress generation hypothesis anticipates that loneliness in one partner may lead to more stressful interactions within relationships, and thus to worse health outcomes for both spouses. This is particularly true among older couples, as life events and shifting time horizons (e.g., retirement, socioemotional selectivity, reduced social networks) lead older persons to focus more time and energy on their closest relationships. Life events such as retirement may make adults’ intimate relationships – and the experiences of their partner – more salient than ever before. In this study, we use dyadic structural equation modeling to examine associations between loneliness and HbA1c levels among 1,331 older married couples from The Irish Longitudinal Study on Ageing. Further, we test whether any such associations vary by age or employment status. Results indicate that one’s own loneliness was not significantly linked with elevated HbA1c, irrespective of age or employment status. However, loneliness of a dyadic partner was significantly associated with elevated HbA1c among retired persons only. Further, this effect was not due to age, but rather to employment status itself. These findings suggest that relationship context is crucial when considering the dyadic health implications of loneliness among the older population.

